# Conquering the stronghold: Membrane‐targeting anti‐infective drug discovery

**DOI:** 10.1002/smo2.70116

**Published:** 2026-10-30

**Authors:** Yanxu Hou, Jiwei Zhang, Bing Huang, Peng Zhan, Xinyong Liu

**Affiliations:** ^1^ Department of Medicinal Chemistry State Key Laboratory of Discovery and Utilization of Functional Components in Traditional Chinese Medicine Shandong Key Laboratory of Druggability Optimization and Evaluation for Lead Compounds Shandong Basic Science Special Academic Zone (Pharmacy) School of Pharmaceutical Sciences Shandong University Jinan Shandong China; ^2^ Institute of Poultry Science Shandong Academy of Agricultural Sciences Jinan Shandong China

**Keywords:** anti‐infective drug discovery, inactivated vaccines, medicinal chemistry, membrane‐targeting strategies, phospholipid flippase

## Abstract

In the realm of anti‐infective drug discovery, traditional paradigms have long been predicated on targeting specific proteins, enzymes, or nucleic acid biosynthetic pathways. However, propelled by the escalating crisis of mutation‐driven resistance, these conventional approaches are currently encountering formidable bottlenecks. Concurrently, despite historical challenges pertaining to selectivity and toxicity, lipid membranes have emerged as validated therapeutic targets in clinical settings. Two seminal studies published in *Nature Communications* (Buchholz et al. 2025) and *Cell* (Chen et al. 2026) have highlighted membrane‐associated mechanisms. Respectively, these studies delineate the application of compound XM‐01 as a broad‐spectrum antiviral agent for the development of inactivated vaccines, and demonstrate that the microbial natural product Butyrolactol A (BLA) potentiates antifungal efficacy via the inhibition of the phospholipid flippase complex Apt1–Cdc50. Focusing on the persistent challenges of viral and fungal infections, this perspective article synthesizes the core findings of these two articles. We systematically elucidated the mechanisms of action underlying compound XM‐01 and the natural product BLA, alongside a critical analysis of their physicochemical properties and pharmacokinetic hurdles. Furthermore, we explore the historical context of membrane‐active agents and outline prospective research directions, with the overarching aim of providing valuable insights and inspiration to investigators in this interdisciplinary field.

## Graphical abstract

This perspective elucidates how XM‐01 and BLA combat infections through modulating pathogen membranes, disrupting biological processes, and potentiating therapeutics, offering cutting‐edge insights into anti‐infective drug mechanisms, innovations, and clinical translation.

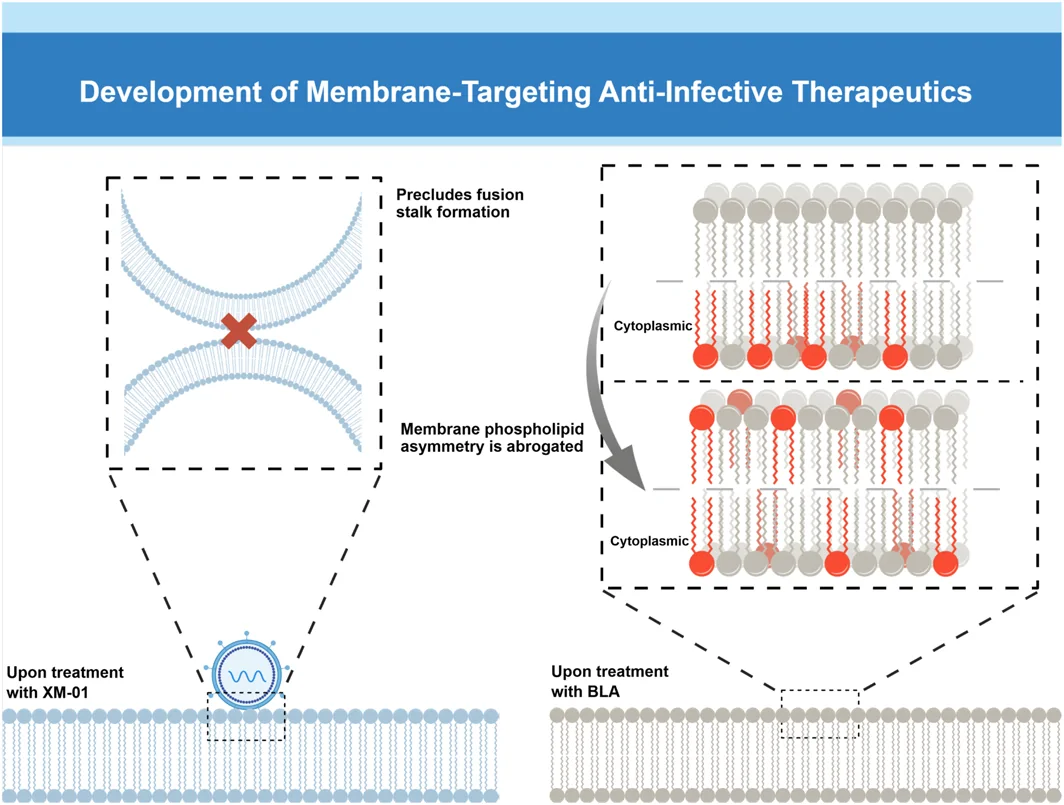

## 1 INTRODUCTION

Pathogenic infections pose a profound threat to global public health, a challenge critically exacerbated by the robust evolutionary adaptability of bacteria, viruses, and fungi.[^1^, ^2^, ^3^] Conventional therapeutics primarily target gene‐encoded proteins or enzymes, imposing prolonged selective pressure that has driven all three domains into an intractable paradigm of drug resistance and immune evasion.[^4^, ^5^] To circumvent resistance induced by single amino acid substitutions, “membrane‐targeting” strategies have long been established as fundamental modalities in clinical practice, particularly within the antifungal field. The lipid bilayer serves as an indispensable physical barrier for pathogen survival. Therapeutics targeting cell membranes have achieved remarkable clinical success. For instance, polymyxins target Gram‐negative bacterial membranes via electrostatic interactions with lipopolysaccharides (LPS),^[6]^ whereas daptomycin, a calcium‐dependent lipopeptide, compromises Gram‐positive bacterial cell membranes.^[7]^ These agents represent crucial “last‐resort” antibiotics. Similarly, amphotericin B remains a pivotal therapeutic for severe fungal infections; its mechanism of action involves direct binding to ergosterol to form transmembrane pores, culminating in ion leakage and subsequent cell death.^[8]^

In the antiviral arena, docosanol acts as a physical blocker directly at the host‐virus interface. Rather than attacking viral replicative enzymes, it alters the biophysical properties of the host cell membrane, thereby impeding the fusion process between the viral envelope and the host cell membrane.^[9]^ Furthermore, arbidol, acting as a multi‐target membrane fusion inhibitor, is utilized for the treatment of respiratory viral infections such as influenza. One of its core mechanisms entails partitioning into the lipid bilayer; by interacting with membrane lipids, it stabilizes the viral envelope, thereby abrogating the fusion of the viral envelope with the endosomal membrane.^[10]^

The application of membrane‐targeting strategies in the anti‐infective field has evolved into a multidimensional research framework. While differences in phospholipid composition between bacterial and human cell membranes can be therapeutically exploited, the development of novel membrane‐active agents—encompassing antifungal peptides (AFPs), their mimetics, and multi‐target membrane disruptors—remains profoundly constrained by a critical bottleneck: selectivity. Because fungal and human cell membranes share fundamental structural architectures, numerous membrane‐targeting candidates have failed in clinical trials due to severe hemolysis or nephrotoxicity.[^11^, ^12^] In the virology domain, small‐molecule inhibitors targeting viral membrane proteins have shown promise. For instance, JNJ‐9676 and CIM‐834, which target the structural membrane (M) protein of SARS‐CoV‐2, exhibit nanomolar potency and have been demonstrated to significantly reduce pulmonary viral titers following oral administration. However, compounded by issues such as the emergence of drug resistance or a lack of broad‐spectrum efficacy, the clinical applicability and safety profiles of these compounds remain limiting factors.^[13]^ Consequently, to address the persistent dilemma of selectivity and toxicity, researchers are currently navigating two divergent trajectories. The first approach involves a paradigm shift in the application scenario: repurposing non‐selective membrane disruptors as tools for in vitro viral inactivation, thereby elegantly circumventing host toxicity. The second approach pursues targeting precision by developing selective modulators capable of specifically recognizing functional intramembrane proteins. Two seminal studies published in *Nature Communications* and *Cell* epitomize this strategic evolution.[^14^, ^15^] The research team led by Hector C. Aguilar repurposed compound XM‐01 to modulate viral membrane fluidity for vaccine development. Concurrently, the teams of Gerard D. Wright and Huilin Li screened approximately 4000 microbial natural product extracts, culminating in the discovery of BLA, a small‐molecule compound that selectively targets the intramembrane flippase complex.

## 2 MECHANISTIC ELUCIDATION

As shown in Figure 1, Aguilar and colleagues have repurposed XM‐01 into a highly efficacious vaccine inactivating agent capable of optimally preserving the native conformation of viral antigens. Mechanistically, XM‐01 deeply intercalates into the lipid bilayer of the viral membrane, increasing membrane “order” and “rigidity,” which significantly elevates its phase transition temperature (*T*_*m*_). These biophysical alterations profoundly inhibit the membrane curvature and deformation required for viral‐host membrane fusion, driving the fusion rate down from a baseline of approximately 21% to roughly 5%, thereby effectively abrogating viral entry. Concurrently, XM‐01 generates perthiyl radicals (RSS•) within the membrane, oxidizing and altering the chemical composition of membrane lipids. Most notably, this induces a dramatic shift in the ratio of phosphatidylcholine (PC) to phosphatidylethanolamine (PE)—from 0.9‐2.0:1 (which is highly favorable for fusion stalk formation) to a highly unfavorable 10.97:1—thereby arresting the formation of the fusion stalk. Crucially, while achieving complete viral inactivation, XM‐01 perfectly maintains the structural and functional integrity of the surface glycoproteins HA and NA. In murine models, vaccines prepared using XM‐01 elicited neutralizing antibody titers and cross‐protective efficacy that surpassed those of traditional formalin‐inactivated vaccines, demonstrating potency comparable to that of live‐attenuated viral vaccines.

**FIGURE 1:**
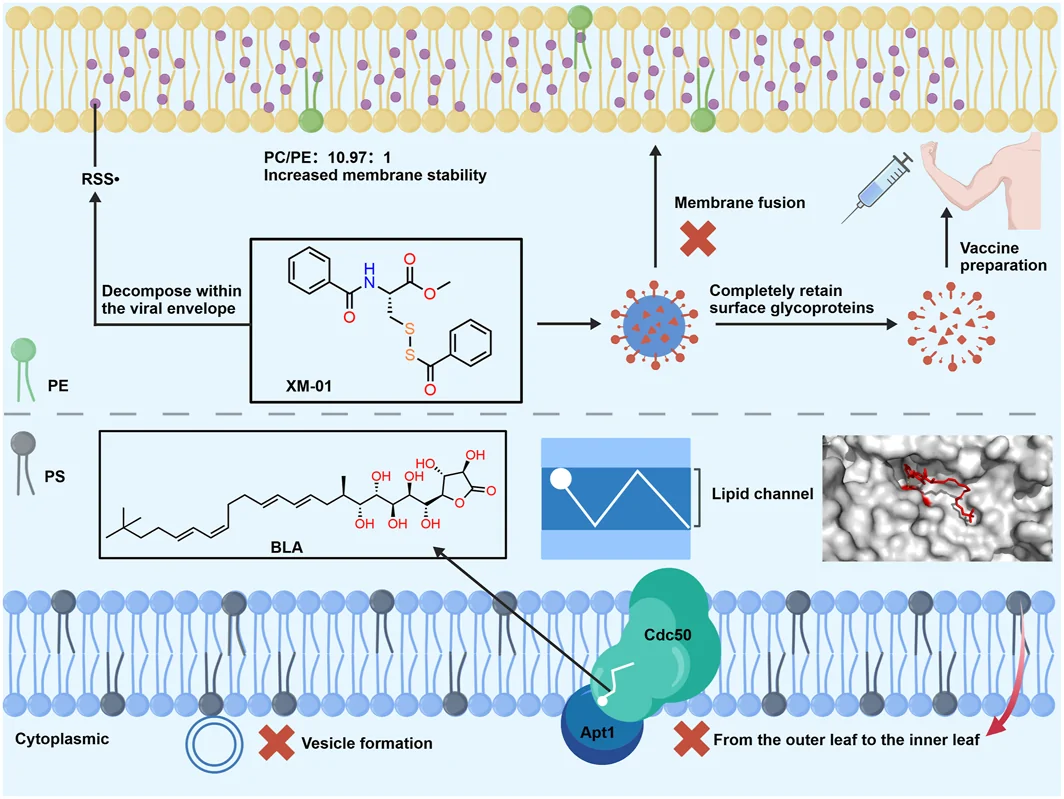
Schematic illustration detailing two distinct mechanistic paradigms for the targeted disruption of pathogenic lipid membrane homeostasis, representing divergent translational strategies: in vitro membrane‐modulating tool versus in vivo targeting of intramembrane protein machineries. XM‐01 abrogates membrane fusion by augmenting viral envelope rigidity via the generation of persulfide radicals (RSS•). This mechanism achieves profound viral inactivation while impeccably preserving the native antigenicity of surface glycoproteins, thereby facilitating the formulation of highly efficacious whole‐virion inactivated vaccines. Concurrently, BLA is precisely anchored within the lipid translocation channel of the fungal phospholipid flippase complex (Apt1‐Cdc50). By exerting potent steric hindrance, it occludes the entry of phospholipid substrates, precipitating a severe compromise in membrane asymmetry. This biophysical destabilization subsequently perturbs global membrane organization, vesicle budding, and intracellular trafficking pathways, ultimately attenuating fungal multidrug resistance. Created with BioGDP.com.^[16]^

In the antifungal field, Wright, Li, and colleagues identified the unique pharmacological properties of the small molecule BLA, which profoundly abrogates the resistance of “superbugs”—particularly *Candida* auris and Cryptococcus neoformans—to the echinocandin drug caspofungin (CAP). Utilizing cryogenic electron microscopy (cryo‐EM), the team revealed that BLA does not engage in simple competitive binding; rather, its hydrophilic head group (a γ‐butyrolactone ring) is precisely positioned at the channel entrance of the fungal plasma membrane phospholipid flippase complex Apt1–Cdc50, while its hydrophobic tail extends into the channel interior. This binding mode firmly locks the complex into an aberrant inactive E2P conformation, aborting its catalytic cycle and lipid‐flipping function. This perturbation triggers a staggering 61% depletion of ergosterol, a critical membrane constituent, leading to aberrant cytoskeletal aggregation in approximately 32% of cells. Ultimately, this structural instability greatly enhances cellular drug uptake, facilitating massive intracellular accumulation of caspofungin. This mechanism enables the eradication of previously intractable lethal fungal infections using sub‐therapeutic doses.

## 3 DIVERGENT TRAJECTORIES IN MEMBRANE TARGETING

The discoveries from both research teams showcase a shared capacity to enhance the efficacy of existing anti‐infective therapies via membrane modulation. However, an in‐depth analysis reveals that they represent two distinctly different developmental trajectories within the membrane‐targeting paradigm: an in vitro physicochemical approach versus an in vivo precise targeting of the intra‐membrane lipid‐protein interface. These two paths illustrate a paradigm shift in membrane‐targeted drug discovery: evolving from the historical “broad‐spectrum, non‐selective membrane disruption” (e.g., early detergents or highly toxic polyenes) to “context‐dependent membrane modulation.” From a pragmatic perspective of clinical translation, we posit that in the near term, there should be a strong inclination toward methodologies utilizing these agents as in vitro vaccine preparation tools. The primary rationale is that employing membrane‐active agents as in vitro processing tools completely circumvents the in vivo toxicity, off‐target effects, and complex pharmacokinetic hurdles that have long plagued membrane‐targeting small molecules. XM‐01, by overcoming the structural degradation caused by conventional chemical inactivators, offers immediate translational value for modern vaccinology. In contrast, while the in vivo pathway represents the ultimate “holy grail” of systemic therapeutics, its trajectory toward clinical approval is much steeper due to inherent druggability limitations.

## 4 FUNDAMENTAL CONSTRAINTS

Despite these breakthroughs, translating these conceptual advances into viable therapeutics requires a critical evaluation of their fundamental constraints. For XM‐01, the challenges extend beyond mere physicochemical optimization; they reflect the systemic limitations of membrane‐active radical‐generating agents. If applied in vivo, such agents fundamentally lack tissue selectivity, posing a severe risk of collateral oxidative damage to host cell membranes. Even in their most optimal application for in vitro vaccine preparation, the fundamental bottleneck lies in the kinetic control of free radical generation. If the release of perthiyl radicals is not strictly self‐limiting, residual radical activity during prolonged vaccine storage will ultimately compromise the very viral surface glycoproteins it initially protected.

Conversely, for BLA, the fundamental constraint is the classical dichotomy between target selectivity and metabolic stability. Although BLA exhibits exquisite structural selectivity at the Apt1–Cdc50 flippase, this high target specificity is insufficient to offset the profound druggability risks imposed by its molecular architecture. The γ‐butyrolactone ring within its structure is highly unstable in human plasma and is exquisitely susceptible to rapid ring‐opening by serum esterases. In an in vivo milieu, the rapid hydrolysis of this pharmacophore would result in premature inactivation before the molecule reaches the site of fungal infection, rendering its high selectivity functionally obsolete.

## 5 SPECIFIC CHEMICAL OPTIMIZATION STRATEGIES

To surmount these systemic and physicochemical bottlenecks, future research must pivot toward specific structural optimization: For XM‐01, future medicinal chemistry efforts must focus on precisely modulating the electronic and steric environments surrounding its sulfur‐containing bond to govern the half‐life of perthiyl radical release. Specifically, introducing electron‐withdrawing groups, such as fluorine or trifluoromethyl substituents, proximal to the sulfur linkage can modulate the bond dissociation energy, thereby fine‐tuning the reaction kinetics. Alternatively, incorporating bulky sterically hindered groups, such as *tert*‐butyl or rigid spirocyclic scaffolds, around the radical‐generating center could prevent runaway oxidative chain reactions. This would ensure that the radicals are sufficiently transient to exclusively inactivate the viral envelope, without lingering and degrading glycoprotein epitopes during the vaccine's shelf life; For BLA: Resolving its metabolic instability is paramount. The vulnerable γ‐butyrolactone ring urgently requires optimization via bioisosteric replacement strategies. Substituting the lactone with a more hydrolysis‐resistant γ‐lactam, cyclopentanone, or a highly fluorinated lactone derivative could effectively shield this pharmacophore from serum esterase‐mediated degradation, while maintaining the critical hydrogen‐bonding network requisite for anchoring to Apt1–Cdc50. Furthermore, because its free hydroxyl group is susceptible to rapid Phase II metabolism (e.g., glucuronidation or sulfation), these metabolic liabilities must be proactively addressed. Finally, to tackle the issue of poor bioavailability, a prodrug strategy could be employed to significantly prolong its systemic circulation time and enhance oral bioavailability.

As our mechanistic understanding of viral and fungal pathogen‐host interactions deepens—particularly regarding membrane‐targeting strategies—the integration of advanced technologies, such as cryo‐EM and AI‐assisted synthetic biology, holds immense promise for propelling the discovery of novel anti‐infective agents and therapeutic modalities.^[17]^ Membrane‐targeting strategies have established a multidirectional R&D landscape across antibacterial, antiviral, and antifungal disciplines, ultimately providing a powerful arsenal against the global public health threats posed by viral and fungal infections.

## AUTHOR CONTRIBUTIONS

Yanxu Hou and Jiwei Zhang drafted the original manuscript. Jiwei Zhang performed the preliminary revisions of the manuscript. Peng Zhan designed the overall writing framework and logical structure of the paper and iteratively revised the manuscript in multiple intermediate versions. Finally, Peng Zhan, Bing Huang and Xinyong Liu completed the final revision of the manuscript.

## CONFLICT OF INTEREST STATEMENT

The authors declare no conflicts of interest.

## Data Availability

Data sharing not applicable to this article as no datasets were generated or analysed during the current study.
